# Examining cross-sectional and longitudinal relationships between multidomain physical fitness metrics, education, and cognition in Black older adults

**DOI:** 10.1080/13825585.2023.2225848

**Published:** 2023-06-22

**Authors:** Matthew Stauder, Kelly J. Hiersche, Scott M. Hayes

**Affiliations:** a Department of Psychology, The Ohio State University, Columbus, OH, USA; b Chronic Brain Injury Initiative, The Ohio State University, Columbus, OH, USA

**Keywords:** Aging, cardiorespiratory fitness, cognition, gait speed, strength

## Abstract

A limited number of studies examine cognitive aging in Black or African American older adults. The purpose of this study was to explore the relationship between health-related fitness metrics, education, and cognition at baseline and over a 4-year follow-up in a sample of 321 Black or African American older adults in the Health and Retirement Study (HRS). Physical fitness was assessed with measures of gait speed, peak expiratory flow, grip strength, and body mass index. Global cognition was assessed with an adapted version of the Telephone Interview for Cognitive Status (TICS). Analyses of relative importance and hierarchical multiple regression were used to examine baseline cross-sectional relationships. Multiple logistic regression was used to examine prospective relationships with longitudinal cognitive status. Education was the strongest predictor of global cognition at baseline and follow-up. More years of education significantly increased the odds of maintaining cognitive status at 4-year follow-up. After accounting for education, gait speed was independently associated with baseline cognitive performance and accounted for additional variance. Grip strength, peak expiratory flow, and body mass index were not significantly associated with cognition. The results indicated that modifiable variables, including years of educational attainment and gait speed, were more strongly associated with global cognition than other modifiable variables including body mass index, grip strength, and peak expiratory flow. The lack of observed associations between other fitness variables and cognition may be attributable to the brief assessment methods implemented, which was necessitated by the large-scale, epidemiological approach of the HRS.

Cognitive decline is common among older adults (Deary et al., 2009). However, considerable individual variability exists in trajectories of cognitive function across the adult lifespan, with accumulating evidence suggesting that modifiable physical fitness variables and sociodemographic factors may impact cognition. For instance, lower levels of cardiorespiratory fitness have been associated with worse performance across multiple cognitive in general cognitive function at a 6-year follow-up among healthy older adults (Barnes et al., 2003). Furthermore, among community-dwelling older adults, sociodemographic variables such as educational attainment have been linked to global cognition (Jefferson et al., 2011). In the absence of a cure for age- and Alzheimer’s disease-related cognitive decline, leveraging the beneficial effects of modifiable variables such as physical fitness and educational attainment could have important implications for the preservation of cognitive abilities with age.

To date, most research examining the relationship between modifiable variables, such as physical fitness and educational attainment, and cognition has been conducted with predominantly White samples (Barnes et al., 2003; Bherer et al., 2013; Hayes et al., 2016). With a racially diversifying population of older adults and the disproportionate incidence of cognitive impairment and dementia specifically among Black or African Americans, there is a clear need to study cognitive aging among more representative samples (Manly & Mungas, 2015). Several well-powered studies have reported that Black older adults presented with lower performance across different cognitive tasks than White older adults (Díaz-Venegas et al., 2016; Weuve et al., 2018; Wilson et al., 2015) but displayed rates of cognitive decline that are similar (Weuve et al., 2018) or slower than White older adults (Wilson et al., 2015). These differences are likely due to an interplay of socioeconomic, psychosocial, and physical health factors. Examples of these factors include disparities in educational attainment, income, perceived discrimination, and prevalence of chronic health conditions (Zahodne et al., 2017), as well as differences in access to educational and health-care resources and potential cultural biases associated with test materials and appropriate normative data (Werry et al., 2019). Notably, a recent study showed that accounting for performance on a multidomain assessment of physical functionality eliminated or attenuated performance differences between Black and White older adults on tasks of global cognitive function, executive function, memory, attention, processing speed, and semantic fluency (Tolea et al., 2020). Additionally, another study found the effect of education on global cognitive performance to be more than twice as large for Black participants compared to White participants (Jean et al., 2019). These studies highlight the importance of considering physical fitness and educational attainment in understanding cognitive performance among Black older adults.

Importantly, physical fitness is a multidimensional construct, and extant research has implicated several domains of physical fitness as relevant to cognition in community-dwelling Black adults. For instance, two studies have shown that peak expiratory flow, which is considered a proxy of aerobic fitness, was linked to global cognition in African American men aged 22–84 years (Sims et al., 2015) and African American older adults (Allaire et al., 2007). In studies with racially diverse cohorts, including sizable samples of Black adults, grip strength, considered a proxy for whole-body strength, appears to have a bidirectional relationship with cognition (George et al., 2021; Leibel et al., 2020). Other studies have shown that faster gait speed was associated with better executive function and semantic memory among Black older adults, controlling for sociodemographic variables, waist circumference, and physical activity (George et al., 2021). Finally, there is mixed evidence for the relationship between body mass index (BMI) and cognition among Black older adults. One study demonstrated that greater changes in BMI was associated with a faster decline in global cognition (Aiken-Morgan et al., 2020), while another reported that Black older adults with extreme values of BMI performed worse on a measure of general cognitive function (Bryant et al., 2014).

To date, there are a limited number of studies that examine the relationship between physical fitness, educational attainment, and cognition in Black or African American older adults. Most extant studies are cross-sectional, limited by sample size, or assess only a single modifiable physical attribute (e.g., gait speed, aerobic fitness, or strength). To enhance our understanding of the relationship between modifiable fitness variables and cognition, it is important to simultaneously assess multiple domains of fitness and their relationship to cognition. For instance, what are the relative contributions of different kinds of fitness (gait speed, aerobic, strength) to global cognition in Black or African American older adults? How do fitness variables compare to more established correlates of cognition, such as education? Which variables are most predictive of maintenance or decline in cognition among Black or African American older adults? To address these questions, we examined cross-sectional and longitudinal associations between multiple modifiable variables, focusing on physical fitness and years of education, and global cognition in a cohort of non-demented, community-dwelling, Black or African American older adults. The study had three main objectives: 1) To explore the relative strength of cross-sectional associations between modifiable variables (gait speed, grip strength, peak expiratory flow, body mass index, education) and global cognition at baseline, 2) To assess whether baseline modifiable variables predict maintenance or decline in cognitive performance over 4 years, and 3) To examine whether rate of change in physical fitness variables was associated with rate of change in global cognition.

## Methods

### Participants

The data were collected from the Health and Retirement Study (HRS) – a biannual nationally representative survey of approximately 20,000 American adults over the age of 50 (Health and Retirement Study, 2022). The HRS is sponsored by the National Institute on Aging (grant number NIA U01AG009740) and is conducted by the University of Michigan. Data from RAND HRS Longitudinal File 2018 (V1) Waves 10–13, taking place from 2010 to 2016, were utilized. Alternating halves of the sample of older adults in the HRS were selected for expanded at-home testing every two years. Therefore, participants repeated testing on an approximate 4-year interval. Older adults aged 65 years or older who self-identified as “Black/African American” and who completed two advanced face-to -face interviews (including physical, health, and cognitive assessments) separated by 4 years were included in the present study. While Black and African American are not synonymous terms and may represent distinct racial identities with unique lived experiences, the HRS did not distinguish between the two in their racial identity classifications. Therefore, for the purposes of this study, we included individuals who self-selected the “Black/African American” response option. All participants provided informed consent before participation in the study.

Individuals with any of the following were excluded from analyses: self-reported history of a physician-provided diagnosis of Alzheimer’s disease or dementia, stroke, psychiatric diagnosis, significant depressive symptom burden defined by endorsing yes on five or more depressive symptom questions from the Center for Epidemiologic Studies – Depression scale and excessive binge drinking (self-reported rate of ≥1 binge-drinking episode per week over the last 3 months). After exclusion criteria were applied, 340 eligible participants with complete demographic, fitness, and cognition data were available for analysis. Statistical outliers (defined as ±3SD from sample mean) on fitness measures (BMI: *n* = 6; peak expiratory flow: *n* = 1; timed walk: *n* = 7) and cognitive outcomes (baseline cognition: *n* = 2; rate of cognitive change: *n* = 3) were removed, resulting in a final analysis sample of 321 Black or African American older adults.

### Measures

#### Physical fitness

Data were collected by HRS personnel during a home visit and included assessments of gait speed, grip strength, peak expiratory flow, and BMI.

##### Gait speed.

Gait speed was assessed with a timed walk task. A 3.7 m walking course was set up in the participant’s home, and interviewers measured the time to walk 2.5 m at the participant’s “normal pace.” The participant completed the task twice. The fastest time in seconds of the two tests was used as the primary metric of gait speed.

##### Grip strength.

Grip strength was assessed twice on each hand using a hand-held Smedley spring-type dynamometer. The maximum value in kilograms across all valid trials for both hands was used.

##### Peak expiratory flow.

Peak expiratory flow (L/min) was measured across three breathing tests with a Mini-Wright Peak Flow meter, and the maximum value was selected.

##### Bmi.

BMI was calculated as the ratio of physically measured weight (kg; Healthometer 830KL scale) divided by the square of height (m^2^; standing against a wall without shoes).

#### Cognition

Global cognitive function was assessed using a modified 12-item version of the Telephone Interview for Cognitive Status (TICS-M; (Brandt et al., 1988)). The TICS-M total cognition score sums the total verbal episodic memory recall (10-word list immediate and delayed recall; 20 possible points) and mental status (full date, day of week, counting backwards starting from 20 and 86, object naming, president and vice-president naming, serial 7 s test, and a 5-word vocabulary task adapted from the Wechsler Adult Intelligence Scale – Revised; 15 possible points) indices, with total scores ranging from 0 to 35.

#### Covariates

Demographic variables included age, gender identity, and years of educational attainment (0–17). Given the disproportionate burden of chronic health conditions on Black and African American populations, we also controlled for a number of chronic health conditions using a health problem index. This self-reported index sums indicator scores for whether a physician has ever diagnosed the participant with the following diseases: high blood pressure, diabetes, cancer, lung disease, heart disease, and arthritis. Possible scores ranged from 0 to 6.

### Data analyses

All analyses were conducted using RStudio Version 4.1.1 (R Core Team, 2022). Independent variables (age, chronic health conditions, education, and fitness measures) and cognitive outcome variables were transformed into z-scores using the mean and standard deviation from the entire sample.

#### Cross-sectional relationships between modifiable variables and cognition

##### Relative importance for linear regression (relaimpo) analysis.

To explore the relative strength of associations between modifiable variables and global cognition at baseline, we used analyses of relative importance – an averaged quantification of regressor contribution to multiple regression models across all possible model orderings – from the *relaimpo* R package (Groemping, 2007). The Lindeman, Merenda, and Gold (lmg) analysis of partitioned variance controls for collinearity and model order to describe the average contribution of each predictor variable to the model outcome. Although the *relaimpo* analysis can identify the relative importance of variables accounting for variance in global cognition, it does not implement significance testing.

##### Hierarchical regression.

To examine whether physical fitness variables accounted for significant variance in global cognition after controlling for other variables, we used hierarchical multiple linear regression as a follow-up to the relaimpo analysis. Order of entry was informed by the results of the *relaimpo* analysis. Age, gender, and chronic health conditions were entered into the model in Step 1. In Step 2, fitness variables with low relative importance on baseline cognition based on *relaimpo* analysis (BMI, grip strength, peak expiratory flow) were entered. In Step 3, years of education was entered. In Step 4, timed walk was entered. This order of entry was chosen for the following reasons: 1) it allowed for the confirmation that grip strength, aerobic fitness, and BMI did not account for significant variance in baseline cognitive performance, 2) it allowed for the assessment of the percent variance in global cognition accounted for solely by years of education after controlling for other variables, and 3) it allowed us to assess whether gait speed (timed walk) significantly accounted for variance after entering more commonly assessed variables such as education into the model.

##### Predicting cognitive status at four-year follow up

To assess whether baseline modifiable variables predict maintenance or a decline in cognitive performance, the sample was divided into cognitive maintainers (those with ≥0 rate of change in cognition, *n* = 152) and cognitive decliners (those with <0 cognitive slope score, *n* = 169). Group differences between maintainers and decliners on modifiable variables were explored using two-sample t-tests. Multiple logistic regression was used to identify which independent variables were associated with maintaining cognitive status at the 4-year follow-up. All variables demonstrating significant group differences were entered into the multiple logistic regression model in one step.

#### Longitudinal relationship between rate of change in fitness and rate of change in cognition

To explore the linear dependence between the rate of change in modifiable variables and the rate of change in global cognition, Pearson correlation tests between fitness variable slope scores and cognitive slope scores were used.

## Results

### Sample characteristics

Descriptive statistics for the final sample are presented in Table 1. 14.0% of participants were from the Northeast United States, 21.5% from the Midwest, 55.8% from the South, and 8.7% from the West. The sample had a mean total wealth, defined as the difference between all assets (e.g., savings and properties) minus debts (e.g., mortgage and loans), of $202,581 (sd = $337,622). 63.9% of the sample reported engaging in moderate intensity physical activity and 40.2% of the sample reported engaging in vigorous intensity physical activity at least weekly.

Black or African American older adult women had significantly higher BMI (29.6 vs 28.3 kg/m^2^, *p* = 0.038), took longer to complete the timed walk (3.7 vs 3.3 s, *p* = 0.005), and had significantly lower grip strength (25.3 vs 39.5 kg, *p* < 0.001) and peak expiratory flow (307.6 vs 397.8 L/min, *p* < 0.001) than their male counterparts. These differences were expected based on previous reports in the literature. There were no significant gender differences in baseline cognitive performance or rate of change in cognition.

### Analyses

#### Cross-sectional relationships between modifiable variables and cognition

##### Relaimpo analysis.

**Figure 1.**
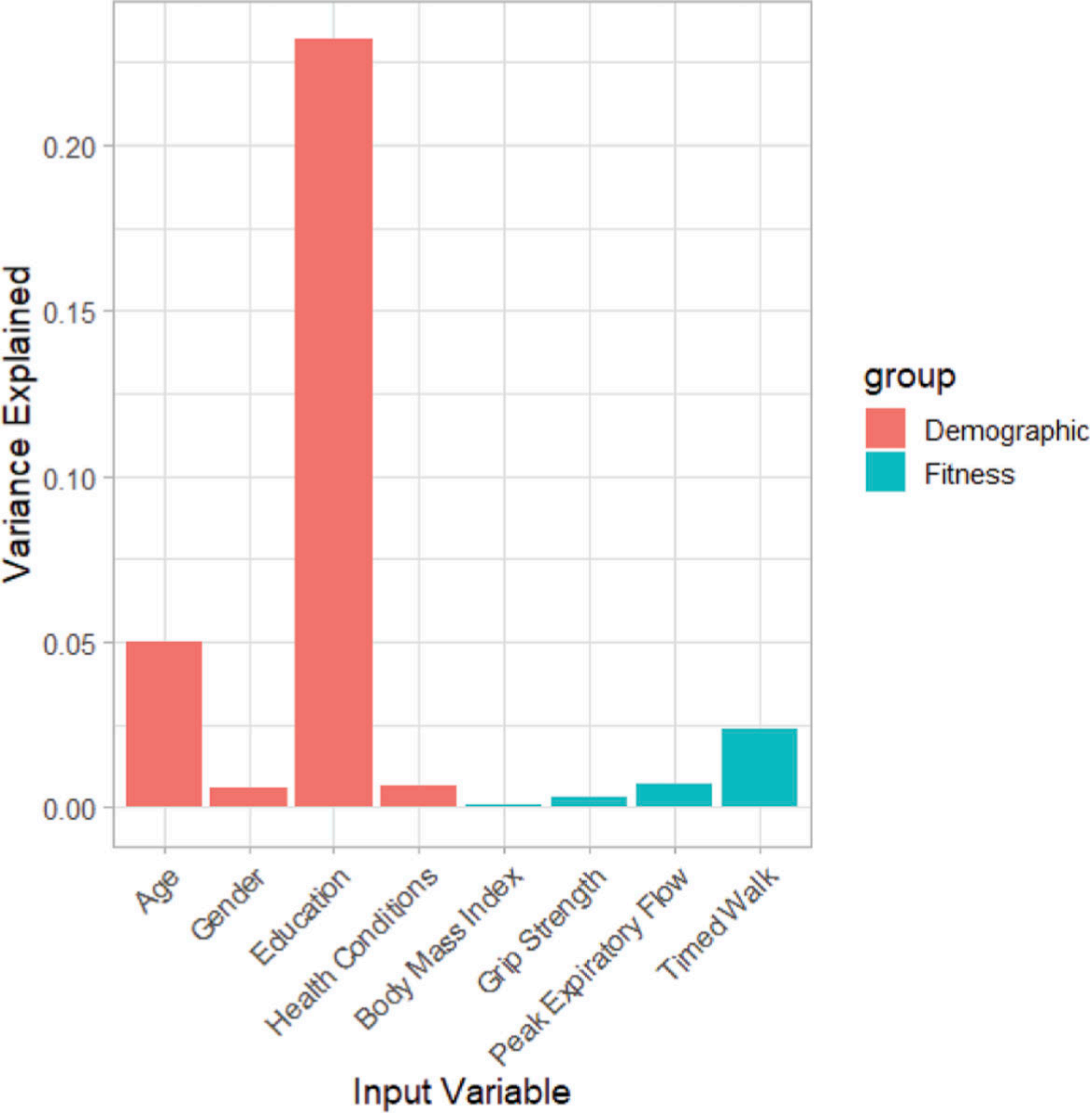
Relative importance of all variables to cross-sectional global cognition.

Relative importance values for all variables included in the cross-sectional model are displayed in Figure 1. Relative importance analyses identified educational attainment as the most robust predictor of global cognition, accounting for 23.2% of variance in baseline TICS scores averaged across all possible model orderings. Age at baseline accounted for an average of 5.0% of variance, followed by gait speed (2.4%) and peak expiratory flow (0.72%). Other variables (gender, chronic health conditions, grip strength, and BMI) exhibited minimal relative importance.

##### Hierarchical regression.

**Figure 2.**
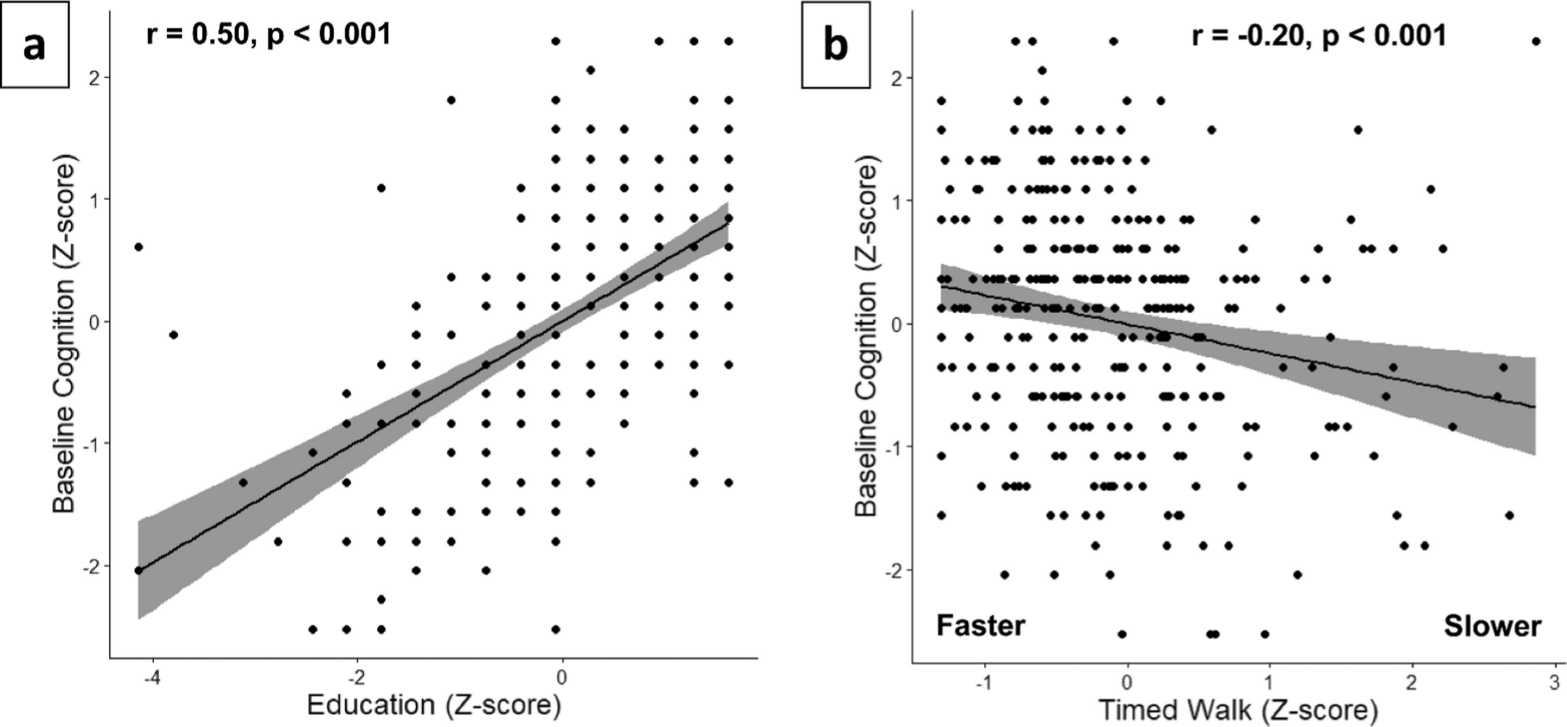
Baseline zero-order correlations. (a) Education and global cognition. (b) Timed walk score and global cognition.

For baseline global cognition, age, gender, and health conditions (Step 1) accounted for 7.3% of the variance and the overall model was significant (see Table 2). Age (β = −0.25, *p* < 0.001) was a significant independent predictor of baseline global cognition. Adding BMI, grip strength, and peak expiratory flow (Step 2) did not improve the model (**Δ**R^2^ = 0.013, *p* = 0.11), although peak expiratory flow was a significant independent predictor (β = 0.12, *p* = 0.046). Adding years of education to the model (Step 3) accounted for an additional 23% of variance in global cognition, which was significant (**Δ**R^2^ = 0.23, *p* < 0.001). In Step 4, adding timed walk improved the model, accounting for a significant, albeit small, amount of variance (**Δ**R^2^ = 0.01, *p* = 0.032). Zero-order correlations between educational attainment and performance on the timed walk with baseline global cognition are displayed in Figure 2.

#### Predicting cognitive status at four-year follow up

Group differences between cognitive maintainers and decliners are presented in Table 1. Of note, there were no significant group differences in any of the fitness variables. When entered into the multiple logistic regression, age (β = −0.34, *p* = 0.007), years of education (β = 0.77, *p* < 0.001), and baseline cognition (β = −0.77, *p* < 0.001) were all significant independent predictors of group status (maintainer or decliner; see Table 3). Older age (OR = 0.71, 95% CI = 0.69–0.91) and higher baseline TICS score (OR = 0.46, 95% CI = 0.33–0.63) were associated with reduced odds of being classified as a cognitive maintainer, while higher educational attainment was associated with increased odds of being classified as a cognitive maintainer (OR = 2.16, 95% CI = 1.59–3.01).

#### Longitudinal relationship between rate of change in fitness and rate of change in cognition

There were no significant relationships between the rate of change in fitness variables and the rate of change in cognition (|r| = 0.01–0.07, p’s = ns).

## Discussion

This study addresses a gap in the literature by examining multiple domains of physical fitness, education, and global cognition in a large sample of Black or African American older adults. Gait speed and years of education were associated with global cognition, whereas peak expiratory flow, body mass index, and grip strength were not associated with cognitive performance. Furthermore, more years of education increased the likelihood of maintaining cognitive performance over a 4-year period. Baseline metrics of physical fitness were not associated with cognitive changes in this sample.

Physical fitness is composed of multiple domains, including motor-performance, cardiorespiratory, muscular, and morphology components assessed in this study (Vanhees et al., 2005). Our finding that a motor component of physical fitness, gait speed, was significantly associated with global cognitive performance is consistent with previous work. For instance, a cross-sectional analysis in a racially diverse sample of older adults found increased gait speed was related to better performance on tasks of executive function, semantic memory, and verbal episodic memory (George et al., 2021). When exploring the influence of race, the association between gait speed and executive function in Black older adults was considerably attenuated but remained significant after adjusting for demographic and socioeconomic variables and cardiovascular risk factors, such as waist circumference (George et al., 2021). This converges with our finding that gait speed was significantly associated with global cognition in Black or African American older adults after adjusting for education, chronic disease burden, and BMI. Importantly, our findings further extend the literature by demonstrating that gait speed was significantly associated with global cognition while also controlling for other fitness-related metrics, such as grip strength and peak expiratory flow. This finding underscores previous research suggesting gait speed may be a crucial independent marker of general health, including cognition. Gait speed may be particularly important for Black or African American older adults. Prior literature has connected reduced speed and other forms of gait dysfunction with global cognitive decline (Cohen et al., 2016; Mielke et al., 2013) and cognitive status in older adults (Callisaya et al., 2017). Cerebrovascular pathology may underpin the relationship between gait and cognition in aging (Cohen et al., 2016). Given the disproportionate prevalence of cardiovascular risk factors (Howard et al., 2017) and cerebrovascular disease among Black or African American individuals (Turney et al., 2023), gait speed may represent an important independent marker of cerebrovascular burden and, in turn, cognitive health among Black or African American older adults.

Our study did not find significant associations between other domains of physical fitness (peak expiratory flow, grip strength, and BMI) and global cognition. Previous studies with Black or African American adult samples have implicated lung functioning, strength, and body mass as important predictors of cognitive ability. Two investigations of peak expiratory flow, a measure of lung functioning, in African American men aged 22 to 84 and African American older adults found peak expiratory flow to be a significant, unique predictor of general cognitive function (Allaire et al., 2007; Sims et al., 2015). These studies used highly similar methods to assess peak expiratory flow (i.e., spirometry) as those implemented in the current HRS sample and controlled for demographic and health-related variables. However, the samples in Allaire et al. (2007) and Sims et al. (2015) were considerably younger, with mean ages of 62.2 and 58.2 years, respectively, and may be less susceptible to survivorship bias. Moreover, the current study used a brief, telephone-based interview to assess cognitive status, whereas the other studies used domain-specific cognitive tasks in addition to brief assessments of cognitive status. It is possible that the telephone-based assessment of global cognitive function used in the current study may be less sensitive to aerobic fitness.

Grip strength, a proxy for whole-body strength, has also been connected to performance across different cognitive domains in older adults. In a study with multiple racial groups of older adults, stronger grip strength was associated with better executive function, semantic memory, and verbal episodic memory, with no significant differences based on race (George et al., 2021). In our study, the zero-order correlation between grip strength and global cognition was not significant. While the sample of Black participants was similar in age compared to our study (mean age of 74.8 years vs 72.8 years), George et al. (2021), utilized a standardized battery of cognitive tests to assess cognition, which may be better suited to capture relationships between grip strength and specific cognitive domains.

Finally, evidence for a relationship between body mass and cognition in older adults has been mixed, including among Black older adults. Some studies have pointed to extreme values of BMI relating to worse general cognitive function among Black older adults (Bryant et al., 2014), while others reported high BMI as protective of cognitive function but changes in BMI related to faster rates of cognitive decline across cognitive domains (Aiken-Morgan et al., 2020). Our results showed no evidence of a significant cross-sectional nor longitudinal linear relationship between BMI and global cognition among Black or African American older adults.

Across the sample, there were significant declines in global cognition, peak expiratory flow, grip strength, and gait speed between baseline and 4-year follow-up. However, longitudinal associations between change in these fitness metrics and global cognition were not observed. We speculate that this could be due to methods implemented in the HRS sample, which were brief in-home assessments that were optimized for a large-scale, epidemiological study rather than gold-standard lab-based assessments. For example, there is evidence that the TICS is a valid screening tool for at-home assessment of dichotomous cognitive status (Knopman et al., 2010), but the measure is constrained by the number and depth of cognitive domains assessed that would be implemented in a lab-based neuropsychological assessment of cognition. Similarly, assessments of physical attributes were optimized for a large-scale epidemiological study but may lack the sensitivity to link to subtle changes in cognition over 4 years in our sample of non-demented older adults. For instance, the gold standard for assessing cardiorespiratory fitness is a progressive, maximal exercise test typically completed on a cycle or treadmill and takes roughly 40 minutes to complete (including setting up cardiac leads, gas exchange, warm-up, and cool-down). This assessment provides multiple metrics of aerobic fitness but would not be feasible to implement in the HRS sample, given the original study goals of HRS.

In contrast to physical fitness attributes, years of formal education was a reliable and robust predictor of global cognition in all models. Education has been considered a proxy for cognitive reserve, which historically has been defined as how flexibly and efficiently one can utilize cognitive and neural resources for a given task and was thought to contribute to the preservation of cognitive abilities and brain health (Stern, 2009). A more recent conceptualization defines cognitive reserve as “a property of the brain that allows for cognitive performance that is better-than-expected given the degree of life-course related brain changes and brain injury or disease” (Stern et al., 2022, p. 101). This definition places cognitive reserve as a component of the broader construct of resilience, or the capacity of the brain to maintain cognition and function with age and insult, along with physiological factors of brain reserve and brain maintenance (Stern et al., 2022). In this updated framework, modifiable variables, including education and physical fitness attributes such as gait speed, grip strength, and aerobic fitness, may contribute to resilience, highlighting the importance of identifying which variables are most strongly associated with cognition.

Although multiple factors such as socioeconomic status, occupation, and engagement in cognitively stimulating activities contribute to cognitive reserve, the strongest proxy for cognitive reserve appears to be years of formal education (Jefferson et al., 2011). However, some studies have reported the persistence of racial differences in performance across cognitive tasks despite matching on years of education (Manly et al., 2002). Factors like education quality, measured via reading level, have been considered an important indicator of neuropsychological test performance among Black or African American older adults (Fyffe et al., 2011; Manly et al., 2002). Given that a standardized measure of reading level was not available within HRS, we utilized years of education as a proxy for cognitive reserve. We observed a strong association between educational attainment and global cognitive performance, with *relaimpo* analyses indicating that education accounted for roughly 23% of the variance on TICS scores when controlling for the order of entry of variables into the model. In the multiple logistic regression model, a one standard deviation increase in years of formal education accounted for a 2.16 times higher odds of maintaining one’s cognitive ability at the 4-year follow-up. These findings underscore the importance of educational attainment to favorable cognitive aging outcomes.

This study had limitations. The assessments of physical fitness and global cognition were suitable for large-scale, longitudinal at-home study assessments but do not represent gold-standard measures of fitness and cognition. Thus, implementation of higher-quality assessments of fitness and cognition may reveal alternative results. However, these gold standard assessments require significantly more resources (e.g., time, money, personnel, equipment, and transportation) and would not have been feasible to implement within HRS study parameters. The HRS also relied on self-report of physician-diagnosed Alzheimer’s disease or dementia. Therefore, it is possible that the sample included individuals with sub-clinical or mild forms of cognitive impairment that were not detected by the TICS. Outliers were removed based on baseline cognitive performance and 4-year rate of cognitive change to minimize the likelihood of capturing individuals at the greatest risk of undiagnosed brain illness or cognitive decline. Similarly, the HRS relied on self-report of medical history, including whether participants had ever been told by a physician that they were diagnosed with high blood pressure, diabetes, cancer, lung disease, heart disease, and arthritis. Therefore, we were not able to objectively verify the status or severity of the reported health conditions.

This study also had multiple strengths. The HRS included a large, longitudinal sample of Black or African American older adults. Metrics from multiple domains of health-related fitness, encompassing morphological, strength, gait, and cardiorespiratory components, were assessed, which is uncommon in the extant literature. Based on inclusion criteria, data from a more representative sample was collected as participants were not excluded due to underlying health or orthopedic issues that would have precluded participation in gold standard fitness assessments. Moreover, assessments were able to be completed in-home by participants, reducing participants’ travel burden. Finally, an exploratory, data-driven approach of using relative importance metrics was used to guide the order of entry into the regression models.

In conclusion, there appears to be a strong link between years of education, a proxy for cognitive reserve, and cross-sectional and longitudinal performance on a telephone-based assessment of global cognition among Black or African American older adults. Among modifiable fitness metrics, gait speed was associated with global cognition, after accounting for multiple sociodemographic variables and other fitness variables. Future research should emphasize gold-standard assessments of fitness and cognition and the recruitment of more diverse, underrepresented groups to further examine relationships between multiple domains of physical fitness and cognitive decline.

## Figures and Tables

**Table 1.** Baseline characteristics of Black or African American older adults (*n* = 321).

Measure (unit)	Mean (SD)	Range	Cognitive maintainers (*n* = 152)	Cognitive decliners (*n* = 169)	*t*-Test (*p*-value)
Age (years)	72.8 (4.8)	65–90	72.4 (4.6)	73.2 (5.0)	1.67 (0.096)
Gender (female, *n* (%))	194 (60.4)	-	96 (63.2)	98 (58.0)	0.69 (0.406)
Education (years)	12.3 (2.9)	0–17	12.8 (2.6)	11.9 (3.2)	−3.00 (0.003)
Number of health conditions	2.1 (1.2)	0–6	2.0 (1.1)	2.2 (1.3)	1.67 (0.097)
Body mass index (kg/m^2^)	29.1 (5.2)	15.2–47.3	28.9 (4.9)	29.2 (5.4)	0.41 (0.68)
Grip strength (kg)	31.5 (9.0)	8.0–55.5	31.7 (9.5)	31.4 (8.6)	−0.32 (0.75)
Peak expiratory flow (L/min)	343.3 (111.2)	90–650	340.6 (109.9)	345.7 (112.7)	0.41 (0.68)
Timed walk (s)	3.5 (1.0)	2.0–7.3	3.5 (1.0)	3.5 (1.1)	0.13 (0.90)
Baseline global cognition	20.6 (4.1)	10–30	20.0 (3.7)	21.1 (4.3)	2.34 (0.02)
Mental status	11.7 (2.4)	4–15	11.7 (2.4)	11.8 (2.5)	0.26 (0.79)
Word recall	8.8 (2.6)	1–18	8.3 (2.3)	9.3 (2.8)	3.45 (<0.001)
Follow-up global cognition	19.8 (4.7)	5–31	21.9 (3.9)	17.9 (4.6)	−8.47 (<0.001)
Mental status	11.6 (2.5)	4–15	12.5 (2.3)	10.9 (2.5)	−6.20 (<0.001)
Word recall	8.2 (2.9)	0–17	9.4 (2.4)	7.1 (2.9)	−7.93 (<0.001)
Annual cognitive slope	−0.2 (0.8)	−2.56–1.96	0.5 (0.5)	−0.8 (0.5)	−23.31 (<0.001)

**Table 2.** Results of cross-sectional hierarchical multiple regression with global cognition as the dependent variable.

Independent variables	Composite global cognition											
	Step 1: Gender, age, and number of health conditions			Step 2: Body mass index, grip strength, and peak expiratory flow			Step 3: Education			Step 4: Timed walk		
	β	t	P	*β*	*t*	*p*	*β*	*t*	*p*	*β*	*t*	*p*
Gender	0.17	1.54	0.13	0.26	1.55	0.12	−0.04	−0.30	0.76	−0.04	−0.29	0.77
Age	−0.25	−4.63	<0.001	−0.23	−4.06	<0.001	−0.24	−4.75	<0.001	−0.23	−4.54	<0.001
Health conditions	0.07	1.34	0.18	0.09	1.63	0.10	0.11	2.35	0.02	0.11	2.26	0.025
Body mass index				−0.06	−0.93	0.35	−0.04	−0.77	0.44	−0.02	−0.44	0.66
Grip strength				−0.01	−0.17	0.86	−0.10	−1.35	0.18	−0.11	−1.52	0.13
Peak expiratory flow				0.12	2.00	0.046	0.08	1.57	0.12	0.06	1.21	0.23
Education							0.49	10.36	<0.001	0.48	10.06	<0.001
Timed walk										−0.13	−2.15	0.032
*R* ^2^	0.073			0.086			0.3196			0.3295		
Δ*R*^2^				Δ*R*^2^ = 0.01, *F*(3, 314) = 2.06, *p* = 0.11			Δ*R*^2^ = 0.23, *F*(1, 313) = 108.61, *p* < 0.001			Δ*R*^2^ = 0.01, *F*(1, 312) =, *p* = 0.032		
Model *F*	*F*(3, 317) = 8.31, *p* < 0.001			*F*(6, 314) = 4.94, *p* < 0.001			*F(*7, 313) = 21.00, *p* < 0.001			*F*(8, 312) = 19.17, *p* < 0.001		

**Table 3.** Results of multiple logistic regression for cognitive slope score.

Independent variable	Binarized global cognition slope			
	Beta	*z*	*p*	OR (95% CI)
Age	−0.34	−2.69	0.007	0.71 (0.69–0.91)
Education	0.77	4.73	<0.001	2.16 (1.59–3.01)
Health conditions	−0.086	−0.71	0.48	0.92 (0.72–1.17)
T1 cognition	−0.77	−4.78	<0.001	0.46 (0.33–0.63)

Predictor variables were standardized. T1 = Timepoint 1.
